# Reconstructive Surgery of Extensive Face and Neck Burn Scars Using Tissue Expanders

**Published:** 2015-01

**Authors:** Mohammad Reza Ashab Yamin, Naser Mozafari, Mohadase Mozafari, Zahra Razi

**Affiliations:** 1 Department of Plastic and Reconstructive Surgery, Shafa Hospital, Kerman University of Medical Sciences, Kerman, Iran;; 2 Department of Plastic and Reconstructive Surgery, 15 Khordad Hospital, Shahid Beheshti University of Medical Sciences, Tehran, Iran;; 3 Medical School, Shahid Beheshti University of Medical Sciences, Tehran, Iran;; 4 Department of Medical Physics, Shiraz University of Medical Sciences, Shiraz, Iran

**Keywords:** Reconstruction, Burn, Scar, Tissue expander

## Abstract

**BACKGROUND:**

Neck reconstruction is considered as one of the most important surgeries in cosmetic and reconstructive surgery. The present study aimed to assess the results of reconstructive surgery of extensive face and neck burning scars using tissue expanders.

**METHODS:**

This descriptive prospective study was conducted on 36 patients with extensive burning scars on the neck and face. Operation for tissue expander insertion was performed and tissue distension started two or three weeks later, depending on the patients’ incisions. After sufficient time for tissue expansion, while removing the expander and excision of the lesion, the expanded flap was used to cover the lesion. Overall, 43 cosmetic surgeries were done.

**RESULTS:**

Rectangular expanders were employed in most patients (73.81%) and were located in the neck in most of them (60.78%). Complications were detected in five patients (13.89%), with exposure of the prosthesis being the most common one. Scar tissues at the reconstruction site and the flap donor site were acceptable in 94.44% and 98.18% of the cases, respectively. Overall, most of the patients (77.78%) were satisfied with the operation results.

**CONCLUSION:**

Using tissue expanders in tissue reconstruction of extensive neck and facial burning scars results in highly desirable outcomes.

## INTRODUCTION

Burn as one of the devastating conditions in emergency medicine can affect all age groups and both genders in all countries while physical and psychological scars and chronic disabilities may be the resultant.^1^ During pregnancy is more important leading to mortality and morbidity in both mother and infant.^2^ The skin of face and neck is commonly exposed to flame burns, boiling water, steam, and caustic agents.^3^ Face and neck reconstruction is considered as one of the most important and most difficult surgeries in cosmetic and reconstructive surgery. However, in burn, there is a great deal of sequella, such as dysfunction and motion limitation of neck, jaws, and lips and growth disorders of the lower jaw due to shrinkage of the skin and scar shortening.^4^

For survivors, the most important problem is scarring with the hope of decreasing the problems related to scar. Wound healing in burn is a complex process of inflammation, granulation, and remodeling of the tissue.^5^ Silver sulfadiazine was shown to be the gold standard in topical burn therapy with antibacterial properties.^6^ There are many reports on resistance of lots of bacteria to silver sulfadiazine.^6^ Therefore, it seems that introduction of new agents for treatment of burn wounds without adverse effects and better efficacy are necessary.^7^ For centuries, the medicinal plants have been extensively used in wound healing of burned injuries.^8^^-^^12^

Cosmetic issue is the chief complaint of these patients.^13^ Thus, the main purpose of reconstruction surgery is replacement of the scar with healthy skin, having good texture, thickness, and color with the most similarity to the area around the scar. In this way, we can maximize the functional and aesthetic outcomes, so that the patients can return to their previous status.^14^ Up to now, several studies have been conducted on the surgical treatment of burn scars on the face and neck, especially in adults,^3^^,^^14^^-^^16^ using skin grafts, locoregional flaps, distant flaps, and free flaps. Among these options, locoregional flaps seem to be the best.^17^

Nonetheless, lack of similar skin is the main problem in these cases. Other problems include facial skin characteristics, its’ anatomical and functional features making it difficult to reconstruct.^18^ Due to the limited adjacent donor tissue to the burn scar area, full-thickness skin grafts and distant flaps are commonly used for repair of extensive scars of the face and neck.^19^ However, because of the obvious differences between the donor and recipient site tissues, these procedures rarely come to desirable results.^20^

Hence, using tissue expanders, delay flaps, and prefabrication have been recommended frequently.^14^ The use of tissue expanders is considered as a choice for head and neck burn reconstruction.^20^ Tissue expander allows the surgeon to repair the extensively damaged areas.^20^ In this technique, the replaced skin has acceptable similarity in color and background to the surrounding skin. In addition, the replaced tissue has a good perfusion. Thus, tissue expanders have become the preferred method in delayed reconstruction of burn scars.^21^

Also, the changes caused by tissue expansion (fat atrophy and thinning of the skin) increase the flexibility of the replaced skin on the face and neck areas making the expanded skin more appropriate than the normal skin.^3^^,^^17^ Furthermore, the use of tissue expansion reduces the donor site morbidity.^22^ Therefore, using tissue expanders, especially in the field of burn surgery, has become an important method of reconstruction in the past 30 years.^23^

In this study, the results of reconstruction of extensive neck and facial scars by tissue expansion have been evaluated. The data of this study were obtained from 36 patients. Since the operation was performed twice in 5 patients and for three times in one patient, a total of 43 operations were taken into account.

## MATERIALS AND METHODS

This prospective study was conducted on 36 patients with extensive post-burn scars on their face and neck areas who had undergone reconstruction surgery from October 2009 to September 2010 in the Department of Plastic and Reconstructive Surgery, 15 Khordad Hospital. The study design and style was approved by Shahid Beheshti University of Medical Sciences Ethics Committee.

In preoperative visits, the donor skin tissue was evaluated to determine the insertion site of the tissue expander, type of the tissue expander, the required dimensions, and the incision site. In fact, the closest expandable tissue to the scar area is considered as the donor tissue. This tissue must have the maximum expandability to replace the burn scar. In case, advance flaps are utilized after selection of the donor tissue, and the incision site is considered where the healthy skin crosses the burn scar. On the other hand, if transposition flaps are employed, the incision site is determined such a way that flap pedicle is not damaged, flap expansion is not disturbed, and the site has the lowest visibility.

Moreover, some points must be taken into account while selection of tissue expanders. First of all, rectangular tissue expanders are believed to have more expandability compared to crescent and round ones. Therefore, the researchers of the present study used rectangular tissue expanders unless their utilization was not possible due to the special form of the scar or restrictions in the donor tissue. 

In this study, non-rectangular tissue expanders were only employed in our cases. Also, the largest possible tissue expanders were used so that they could be located under the donor tissue. In this study, the patients took a shower the night before the surgery. A prophylactic antibiotic was prescribed for all the patients during tissue expander insertion and removal procedures. Cefazolin was intravenously used at the dose of 1000 mg in adults and 500 mg in the children over 5 years old (0.5-1 hour before the surgery) and was continued for 24 to 72 hours after the surgery.

Tissue expander insertion was performed under general anesthesia. At first, the skin was prepared with scrub bethadine and then it was colored with green bethadine. Adrenaline solution from 1/100000 to 1/200000 was used in the incision site and the pocket area and then, the subcutaneous pocket was dissected in all areas (subgaleal pocket in scalp area). Fine hemeostasis and washing of the pocket area was performed using normal saline. Afterwards, the tissue expander was inserted in the pocket. 

Before inserting the tissue expander, it was washed with antibiotic solution (500 ml normal saline and 160 mg gentamicin) and examined in terms of safety and leakage. During the insertion procedure, knuckling or bending of the prosthesis was avoided. The pocket was designed 10-20% larger than the tissue expander below the donor tissue. This flap was designed subcutaneously and on the surface fascia. The injection port was placed under the healthy skin in a location far from the pocket and the tissue expander. The location of the port must be under the subcutaneous tissue, accessible for the following injections, easily distinguishable, and far from bone tuberosity. It should be mentioned that tension, kinking, and rotation should be avoided during port insertion; otherwise, unsuccessful expansion would possibly take place. Also, port performance should be examined while operation. 

A vacuum drain was also placed in the site if necessary. Vacuum drains were used in case of uncertainty about hemostasis or probability of fluid accumulation at the pocket site, for instance due to adjacency to cervical lymph nodes. Then, the fascia and the subcutaneous area were repaired using vicryl suture and the skin was closed using nylon 6/0. As required, normal saline was injected as much as ten to twenty percent of the volume of the tissue expander or more in a way that not much tension was applied to the skin covering the tissue expander and the repaired wound. After the surgery, the wounds were dressed using sterile gauze.

Tissue expansion began within two or three weeks, depending on the wound condition; i.e., when the wound was completely closed and had no signs of inflammation or infection. After sufficient tissue expansion, the tissue expander was removed and the scar area was excised. Tissue expansion was measured by subtracting the tissue expander’s base size from the width of the skin on the exposed tissue expander. This measure must be at least 10-20% more than the scar width, so that sufficient skin is created for covering the resultant defect while tissue expander removal. After that, the scar area was covered by the expanded skin under general anesthesia. The incision site was repaired in two layers. 

After the surgery, all the patients were analyzed and compared in terms of demographic variables, such as age and gender, cause of scar, location of scar, the largest and smallest lesion diameters, prosthesis shape, tissue expander insertion site, the mean initial volume and final volume of the prosthesis, the mean interval between implant placement and reconstructive surgery, complications of the prosthesis, color homogeneity, flap thickness and its consistency with the surrounding skin tissue, complications and scar formation in both reconstructed area and flap donor site, and patient satisfaction. 

For interpretation of the quantitative variables, mean, standard deviation, minimum, and maximum values were used. For interpretation of the qualitative variables, on the other hand, absolute frequency and relative frequency were used. All the analyses were performed using the SPSS statistical software for windows (version 17.0 SPSS Inc., Chicago, IL, USA). After all, three specialists evaluated the successfulness of the operation regarding replacement of scar with healthy skin, homogeneity of the replaced skin with the surrounding skin tissues, tissue thickness, and normal performance of the tissue at least 3 months after the reconstruction surgery. All the patients with face and neck scars with surrounding healthy tissues for expansion and scar coverage were enrolled into the present study.

## RESULTS

**Fig. 1:**
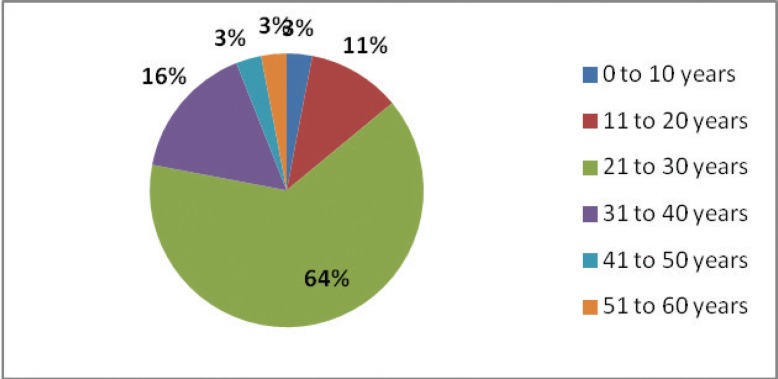
Frequency distribution of all age groups of the patients

The clinical findings of 43 operations of 36 patients were enrolled. The mean age of the patients was 26.97±8.18 years and 66.7% of them were female. The frequency distribution of age groups has been listed separately in Figure 1. 

The mean diameters of the largest and smallest lesions were 18.48±6.19 and 13.11±6.07 cm, respectively**. **In addition, the mean initial volume of the prosthesis was 321.55±182.52 ml that was brought to the final volume of 865±623.64 ml**. **Besides, the mean volume of the expanded prosthesis was 552±526.73 ml (minimum: 95 ml and maximum: 3000 ml). Moreover, the mean interval between implant placement and reconstruction surgery was 190.74±66.88 days. The results of frequency distribution of the patients’ characteristics of lesions and tissue expanders are presented in Table 1. Also, frequency distribution of the patients according to the results of surgery and cosmetic variables is shown in Table 2.

**Table 1:** The distribution of patients by characteristics of the lesions and tissue expanders

**Variable**	**Absolute frequency**	**Relative frequency (%)**
Cause of scar		
Fire burns	20	60.61
Boiling water burns	5	15.15
Acid burns	1	3.03
Other burns	2	6.06
Accident	3	9.09
Iatrogenic	2	6.06
Location of scar		
Face	27	54.00
Forehead	4	8.00
Neck	18	36.00
Shoulder	1	2.00
Prosthesis shape		
Crescent	5	11.90
Cylindrical	1	2.38
Rectangular	31	73.81
Round	5	11.90
Insertion site of tissue expander		
Face	6	13.04
Forehead	3	6.52
Neck	28	60.87
Shoulder	5	10.87
Scalp	2	4.35
Chest	2	4.35

**Table 2:** The distribution of patients’ by characteristics of surgical outcome and cosmetic variables

**Variable**	**Absolute frequency**	**Relative frequency (%)**
Assimilation of flap’s color with the surrounding skin		
Very good	14	38.89
Good	14	38.89
Moderate	8	22.22
Assimilation of flap’s consistency with the surrounding skin		
Very good	18	50.00
Good	15	41.67
Moderate	13	8.33
Assimilation of flap’s thickness with the surrounding skin		
Very good	15	41.67
Good	14	38.89
Moderate	7	19.44
Scar formation in reconstruction site		
Acceptable	34	94.44
High	2	5.56
Scar formation in flap donor site		
Acceptable	31	91.18
High	3	8.82
Overall patients satisfactory by operation results		
Very good	10	27.78
Good	18	50.00
Moderate	7	19.44
Low	1	2.78

**Fig. 2:**
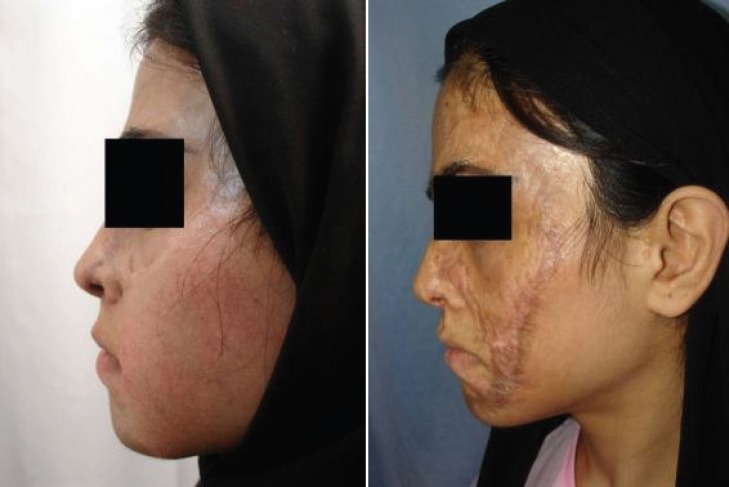
The first patient before operation (Right) and after operation (Left).

**Fig. 3:**
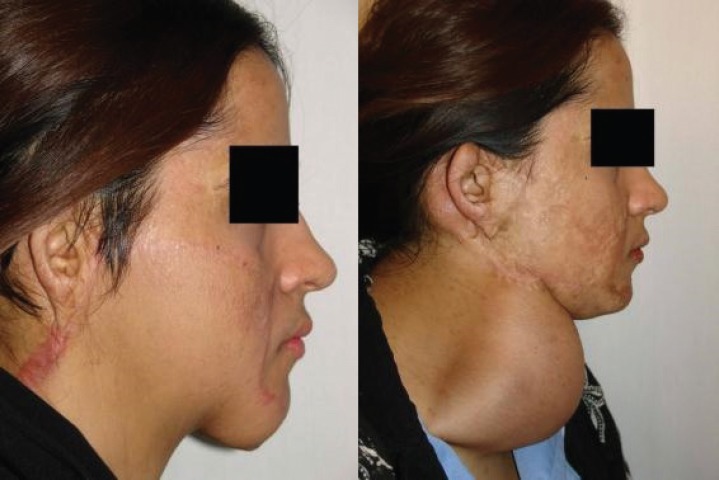
The second patient before operation (Right) and after operation (Left).

**Fig. 4:**
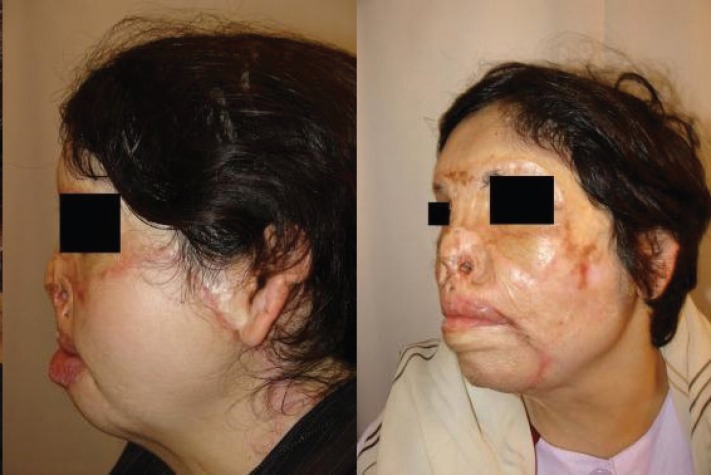
The second patient before operation (Right) and after operation (Left).

**Fig. 5:**
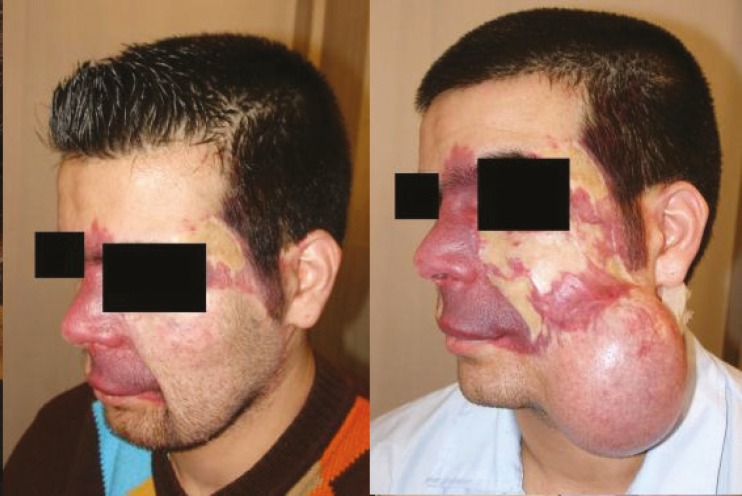
The second patient before operation (Right) and after operation (Left).

**Fig. 6:**
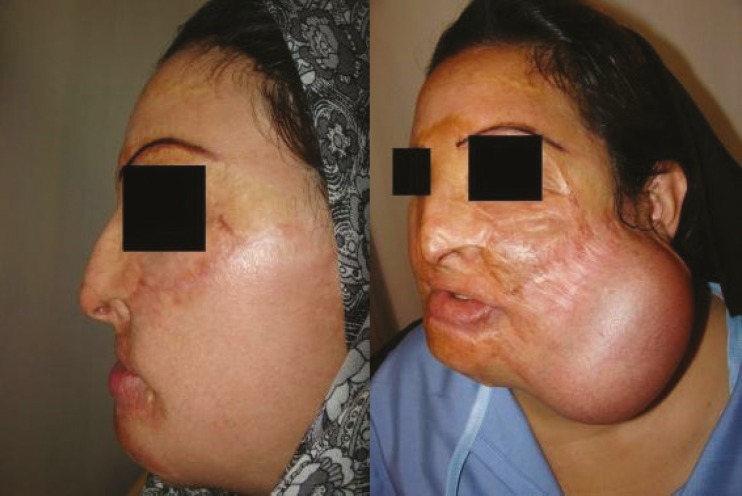
The second patient before operation (Right) and after operation (Left).

**Fig. 7:**
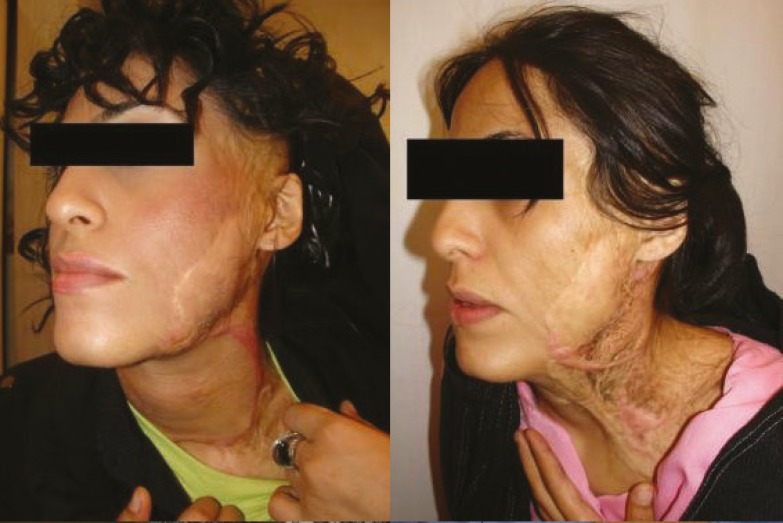
The second patient before operation (Right) and after operation (Left).

**Fig. 8:**
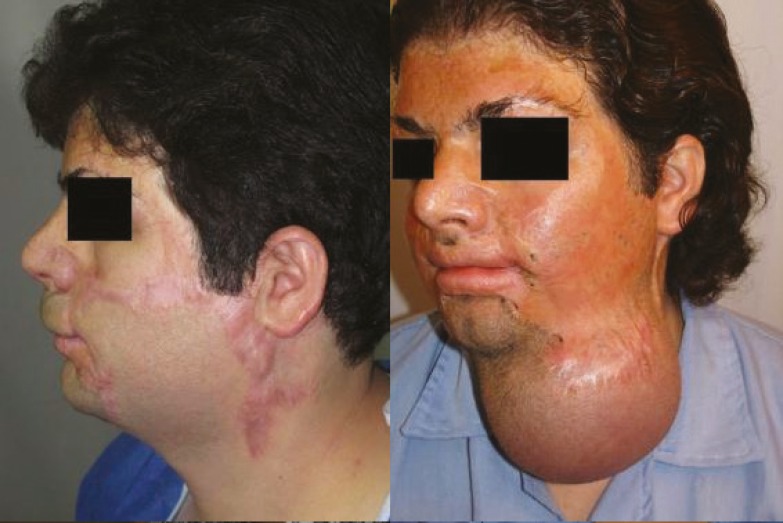
The second patient before operation (Right) and after operation (Left).

It is worth mentioning that the prosthesis related complications were only detected in 5 patients (13.89%), including prosthesis exposure (2 cases, 5.56%), prosthesis site infection (1 case, 2.78%), leakage of the prosthesis (1 case, 2.78%), hematoma (1 case, 2.78%), and necrosis of injection port site (1 case, 2.78%) which led to extraction of the port (the external port) and repair of the initial location of the port. Besides, the complications related to the reconstructed skin were observed in 5 patients (13.89%), including wrinkling (3 cases, 8.33%), inadequate coverage (3 cases, 8.33%), and superficial necrosis (2 cases, 2.78%). Overall, most of the patients (77.78%) were satisfied with the operation results. As shown in Figures 2 to 8, reconstruction of the face and neck scars using this method not only have been very pleasant in terms of the beauty for both patient and his/her family, but also it has shown a significant improvement on the patient’s appearance.

## DISCUSSION

Based on the results of this study, the mean age of the patients was 26.97±8.18 years. Besides, 24 patients (66.67%) were female. In the study by Motamed *et al.* also, the mean age of the patients was 25.5±8.3 years and the majority of them (70.59%) were female.^24^ In another study by Gao *et al.*, 57.17% of the patients were female.^25^ Farahvash *et al.* also came to similar results.^26^ The cause of scar was fire burn (60.61%) followed by boiling water burn (15.15%) in most of the cases. Similarly, Motamed *et al.* showed that the most common cause of burn was flame (47%) and boiling water (26.5%).^24^ Our study results indicated that the scars were mostly located on the face (54%) and the neck (36%). Consistently, Motamed *et al.* reported that the scars were mostly located on the face (70.59%) and the neck (23.53%).^24^

Selection of size, shape, and location of tissue expander and location of injection port is very important. Motamed *et al.* used rectangular tissue expanders and claimed that using these expanders might increase the options for flap design.^20^ Rectangular shaped prosthesis was used in the majority of the patients in this study (73.81%)**. **Motamed *et al.* in another study showed that rectangular prostheses were used in 58.82% of the cases.^24^

In this study, the mean initial volume of the prosthesis was 321.55±182.52 ml that was brought to the final volume of 865±623.64 ml**.** Therefore, the mean of prosthesis expansion was 552±526.73 ml (minimum: 95 ml and maximum: 3000 ml)**. **Gao *et al.* showed that the volume of prosthesis expansion was between 800 and 1200 ml.^25^ In another study, the initial volume was selected between 250 and 500 ml.^24^

In general, tissue expanders are placed in the regions near the scar on the face, neck, or scalp which have the most similarity with the skin color.^20^ Thus, reconstruction of skin scars with these areas is highly desirable.^17^ Based on the present study results, neck was the most common site for expander placement (60.78%). Previous studies have also shown that the healthy skin of the neck has the maximum similarity with the facial skin**. **Areas around the neck, such as shoulders, scapula, and pectoralis region, are also similar to the neck.^15^

In the current study, complications of prosthesis were detected in 5 patients (13.89%), including prosthesis exposure (5.56%), prosthesis site infection (2.78%), leakage of the prosthesis (2.78%), hematoma (2.78%), and necrosis of the injection port site (2.78%). In the study by Bozkurt *et al.*,^21^ the complication rate was estimated to be approximately 30%. Other studies have reported 1.22%,^20^ 4%,^21^ and 5%^18^ of infection rates. According to Spence *et al.*, the patients’ complications included infection (5%), exposure of tissue expander (3%), tissue expander or injection port malposition (3%), shrinkage of tissue expander (7%), and loss of a part of the flap (3%).^18^ Moreover, Kawashima *et al.* mentioned the complication rate to be 34.8%, including prosthesis exposure (13%), tissue expander laceration (8.7%), hematomas (8.7%), flap necrosis (4.3%), and infection (4.3%).^27^ Furthermore, Chung and colleagues reported a complication rate of 12% in their research.^28^ In the study conducted by Motamed *et al.*, the only problem was epidermolysis of the distal flap which was seen in 2 patients who were treated by medical therapy.^24^

The present study also showed that the complications of skin reconstruction were detected in 5 patients (13.89%), including wrinkle (8.33%), superficial necrosis (2.78%), and inadequate coverage (8.33%). Nevertheless, 98.18% of the flap donor site scars and 94.44% of the reconstructed site scars were acceptable. Meticulous and careful planning for using flap donor while application of tissue expanders leads to a reduction in the complications and face deformities. Standardization of preoperative care and placement, expansion, and reconstruction techniques will reduce the rate of the subsequent complications, as well.^20^

Regarding the use of tissue expanders, the reconstructed flap texture and color should be in good assimilation with the recipient area.^25^ In this study, the assimilation of the flap’s color (77.8%), consistency (91.67%), and thickness (80.56%) with the surrounding skin was desirable in most of the cases (Tables 1 and 2).

Ultimately, a study showed that most patients (77.78%) were satisfied with the operation results. Feng *et al.* showed that the use of ultra-thin cervico-pectoral flaps using tissue expanders for complete reconstruction of the anterior neck was associated with excellent results in 4 and good results in 2 patients.^29^ Motamed and colleagues also showed that both patients and the surgeon were satisfied with the operation results.^24^ In another study, the results of reconstructive surgery were quite satisfactory in all the patients.^20^

One of the limitations of this study was the impossibility of following some patients up after the surgery. Also, because many patients were referred from a distant city, sometimes the injection interval became too long. Overall, the results of this study has shown that using tissue expanders in reconstruction of extensive face and neck burning scars has lead to satisfactory results. As shown in Figures 2 to 8, reconstruction of the face and neck scars using this method not only have been very pleasant in terms of the beauty for both patient and his/her family, but also it has shown a significant improvement on the patient’s appearance. Yet, more research is required to investigate some methods for lessening the complications of these techniques. 

## Note

Please cite this paper as:

Ashab Yamin MR, Mozafari N, Mozafari M, Razi Z. Reconstructive Surgery of Extensive Face and Neck Burn Scars Using Tissue Expanders. World J Plast Surg 2015;4(1):40-49.
